# Non-academic factors influencing the development of empathy in undergraduate nursing students: a cross-sectional study

**DOI:** 10.1186/s12912-021-00773-2

**Published:** 2021-12-08

**Authors:** Nancy Berduzco-Torres, Pamela Medina, Montserrat San-Martín, Roberto C. Delgado Bolton, Luis Vivanco

**Affiliations:** 1 Universidad Nacional San Antonio Abad del Cusco, Av. de La Cultura 773, 08000 Cusco, Peru; 2 Universidad de Granada, C/ Santander 1, 52005 Melilla, Spain; 3 Hospital Universitario San Pedro, C/Piqueras 98, 26006 Logroño, Spain; 4 Centro Nacional de Documentación en Bioética, C/Piqueras 98, 26006 Logroño, Spain; 5 Centro de Investigación Biomédica de La Rioja (CIBIR), C/ Piqueras 98, La Rioja 26006 Logroño, Spain

**Keywords:** Nursing students, Empathy, Loneliness, Personality development, Sex characteristics, Universities

## Abstract

**Background:**

Empathy is described as a core competence of nursing. There is abundant research evidence supporting that empathy varies according to personal characteristics and targeted training. The aim of this study was to characterize non-academic factors (personal and environmental) influencing the development of empathy in undergraduate nursing studies who are not receiving a targeted training in empathetic abilities in their nursing schools.

**Methods:**

A cross-sectional study was performed in the three nursing schools located in Cusco city, Peru (two private and one public). The Jefferson Scales of Empathy, Attitudes toward Physician-Nurse Collaboration, and Lifelong Learning, the Emotional Loneliness Scale for Adults, and the Scale of Life Satisfaction, were applied as the main measures. Also, information regarding gender, nursing school, and age, were collected. After psychometric properties were assessed, all measures were used in the development of a multivariate regression model to characterize factors of influence in empathy.

**Results:**

In a sample composed by 700 undergraduate nursing students (72 males and 628 females), a multivariate linear regression model was created. This model explained the 53% of variance of empathy and fitted all conditions necessary for inference estimations. Teamwork abilities, loneliness, age, sex, subjective well-being, and nursing school, appeared as factors influencing the development of empathy in patients’ care.

**Conclusions:**

Findings have indicated that, in absence of a targeted training, individual characteristics and characteristics associated with social and family environments play an important role of influence in the development of empathy in nursing students. These findings are also in consonance with others previously reported in different cultural settings including high-, middle- and low-income countries.

**Supplementary Information:**

The online version contains supplementary material available at 10.1186/s12912-021-00773-2.

## Introduction

Empathy has been described as an important component of professionalism in healthcare and especially in those disciplines that are in direct contact with patients, such as nursing [1]. In fact, it is widely accepted that the ability of nurses to empathize with their patients is a desirable quality, and that patients want empathic and emotionally competent nurses. Moreover, there is evidence that empathy enables nurses to handle difficulties better [2]. According to some authors [3], nurse educators play an important role of responsibility as providers of an education that engenders empathic understanding. In consonance with this, Richardson and colleagues have suggested the inclusion of targeted training in academic programs, such as “nursing therapeutics” methodology, teaching nursing students how to care using compassion and empathy [4]. In recent years, similar training experiences based on communication and understanding skills have demonstrated a positive effect in the enhancement of empathy in medical [5, 6] and nursing students [7, 8].

However, there are still many countries in which targeted training focused on acquiring and improving empathic abilities is a pending task [5, 6, 9]. In the specific case of Peruvian medical and nursing schools, targeted training programs focused on the acquisition of empathic abilities are included in their curricula in a few number of cases [6]. In the majority of cases, this type of knowledge is expected to be acquired as part of the clinical training that medical and nursing students receive in advanced stages of their studies. Recent studies have highlighted that the lack of appropriate support, role modelling, and focused training in Peruvian institutions is associated not only with no improvement in empathy, but also with a deterioration of other aspects such as ethics and emotional wellbeing [9, 10]. Under these circumstances, it is clear that personal skills and non-academic environments (such as family and cultural ones) have acquired more relevance as possible main sources of influence in the development of empathic abilities in nursing students, especially when targeted training activities on empathetic abilities are missing or limited.

## Background

When referring to clinical interactions, empathy has been described as a professional competence that is principally cognitive, more than affective or emotional. Such distinction between cognition and emotion (and correspondingly, between *empathy* and *sympathy*) may not seem as important in situations different from clinical work, in which both elements could have a similar importance [11]. However, in clinical encounters, establishing a relationship with the patients mainly based on a cognitive or an emotional response can derive in different outcomes for patients and for healthcare professionals [12]. In clinical encounters, cognitive mental processing primarily refers to an advanced intellectual process that often involves social perception, analysis of information, and generation of appropriate responses based on the ability to understand patients’ concerns, which are some of the main components of clinical empathy. In contrast, an emotional response consists primarily in a more primitive mental process, where the response is consequence of an affective resonance in healthcare professionals of the emotions observed in their patients, which is a characteristic element of a sympathetic reaction. According to Hojat [13], “the distinction between sympathy (also known as emotional empathy, or vicarious empathy) and empathy (also known as cognitive empathy, or clinical empathy in the context of patient care) has important implications for the clinician–patient relationship because joining the patient’ emotions, a key feature of sympathy, can impede clinical outcomes”. In this sense, it could be recommended for a healthcare professional to sense the patient’s feelings only if it does not impede his/her professional work [14]. Otherwise, it can be difficult to keep a sense of whose feelings belong to whom [15]. Fernandez and Zahavi recently stated in this matter, “Empathy in its most basic sense offers a direct and immediate form of other-understanding–one that doesn’t require us to reproduce or share the other’s experience” [12]. Without concluding that emotions are, *per se*, a barrier in the establishment of an empathic relationship with patients, their role is subordinated under the control of cognitive components. Studies on social-neuroscience recently have shown evidence demonstrating that an affective sharing may act as a gain antecedent to empathic understanding, while cognitive components are important for representing the mental states of self and other, necessary to make decisions in a clinical context [16].

On this basis, there are three elements that are generally accepted as components of empathy in clinical contexts [13]: (i) comprehending patients’ experiences, concerns and perspectives; (ii) a good and clear communication; and (iii) an intention to help, expressed in a compassionate (benevolent) attitude aimed at taking care of a sick person. The first two components, mostly described as “mind’s eye” (understanding) and “third ear” (listening), have been associated with socio-emotional processes in experimental studies, such as social knowledge, social perception, and decision-making [17, 18]. These two components are constitutive of the “empathic curiosity” that, according to Jodi Halpern, encourage an empathic response in clinicians focusing on learning more about what their patients are experiencing and how it affects their attitude towards their treatment [11, 19]. The third component of an empathic response, “intention to help”, has been associated with the capability of controlling personal anxiety derived from the exposition to patients’ suffering [20]. Neuroimaging studies suggest that in the background of this emotional regulation there is a neural control of brain regions involved in emotional responses, such as the insula, the anterior cingulate cortex, and the periaqueductal gray [16, 21, 22]. When focusing on nurses, an intention to help as the main personal interest has been described as an attribute that allows them to perceive the patient as like self while keeping a clear separation between self and the patient [13]. In accordance with this, much of the nursing literature suggests that nurses should not rely on their innate capacity for emotional empathy; instead, they should develop intellectual techniques for understanding their patients in an objective manner that provides distance from their patients’ emotional distress [12]. From a phenomenological perspective, some authors complete this approach with the consideration that nurses, based in the altruistic nature of nursing, have more resources to be open to and appreciate the individuality of each patient in their daily work [23].

Regarding possible individual factors influencing the development of empathy in healthcare professions, findings from a large number of studies suggest that women are often more empathic than men, obtaining higher scores in empathy measures [24–27]. These findings are in consonance with others performed in general population where women have shown better indicators in neurological measures related to empathy [28]. Certain genetic predisposition, evolutionary underpinnings, and interpersonal styles, but also social interactions have been described as possible explanations for such gender differences in empathic responses [29]. In medical students, personality [30], personal motivation for studying medicine [31], and career interest [24] have also been described as other influencing factors in the development of empathy.

In relation with the role that the environment plays in the development of empathy, an increasing number of studies have reported evidence supporting its influence at family, social, and cultural levels. In the general population, individuals from communities with greater prosocial indicators, such as higher well-being and higher volunteering rates, have shown higher scores in empathy measures in comparison with those who were living in communities where violence and crime rates were higher [32]. In consonance with this, cross-cultural studies performed with healthcare professionals [33] indicate that culture plays a role of influence in the empathic response to the patients. Studies with medical and nursing students [34–36] also suggest similar findings associated with certain social and cultural environments. Furthermore, studies performed in the United States [37] and recently in Peru [38] have reported that parents and family environments play a role of influence in the development of empathy in medical students.

Taking this into account, this study was designed with the purpose of testing the following hypothesis: In the absence of a targeted training in empathy, its development in nursing students is influenced by individual characteristics and by the influence of the social and family environments. Three objectives were established with this purpose: (i) to identify differences in empathy according to sex and nursing schools groups; (ii) to analyse the type of association existing between empathy and other two professional competences: inter-professional collaboration and lifelong learning abilities; and between empathy measures and students’ wellness self-perception (subjective well-being), students’ perception of their parents (family environment), academic achievement, and perception of loneliness; and (iii) in those cases in which differences were confirmed, to characterize factors influencing empathy’s measurements.

## Methods

### Design and participants

In the second semester of 2019, a cross-sectional study was carried out in the three nursing schools, two private and one public, located in Cusco city in Peru. None of these institutions offered in their curricula a specific course or training program (neither mandatory or elective) focused on empathy or communication and understanding abilities in patient care.

The entire population of undergraduate students enrolled in these institutions was 1030 students. However, for the purpose of this study only undergraduate students attending academic activities in Cusco city were included. Those who were attending academic activities elsewhere when this study was performed, such as communitarian work in isolated rural communities, exchange and internship programs in other institutions, were excluded. Students’ participation was voluntary and anonymous. On this basis, the estimation of the minimum sample size required has been calculated with the G*power software, version 3.1.9.7. For this calculation, it was expected to create a regression model based on a linear multiple regression analysis with an effect size between small and medium (Cohen-*f*^2^=0.085), an alpha equivalent to 0.05, a power of 0.95, and at least 5 tested predictors from 10 variables analysed. It was also assumed a 25% of missing questionnaires (questionnaires that were partially answered). According to this analysis, the minimum sample size required was 319 participants.

### Measures

For measuring empathy, the Healthcare student’s version of the Jefferson Scale of Empathy (JSE-HPS), was used. The JSE-HPS (20 items) is answered in a Likert scale from 1 (*strongly disagree*) to 7 (*strongly agree*). The JSE-HPS follows the same structure of the medical student’s version of the JSE (JSE-S). The main difference between both versions is the rewording of terms “medicine” or “physician” to make it clearer for students from healthcare areas different than medicine [13].

To measure inter-professional collaboration (teamwork) between nursing and medicine, the Jefferson Scale of Attitudes toward Physician-Nurse Collaboration (JSAPNC) was used [39]. The JSAPNC responds to the definition of teamwork as an ability of nurses and physicians to work together cooperatively, sharing responsibilities for solving problems and making decisions to formulate and carry out plans for patient care. The JSAPNC (15 items) is answered using a Likert scale from 1 (*strongly disagree*) to 4 (*strongly agree*).

The Healthcare student’s version of the Jefferson Scale of Physician Lifelong Learning (JeffSPLL-HPS) was used to measure attitudes towards lifelong learning [40]. The JeffSPLL measures the development of skills related to information gathering, the use of learning opportunities, and self-motivation [41]. The JeffSPLL (14 items) is answered using a Likert scale from 1 (*strongly disagree*) to 4 (*strongly agree*). Similarly to the JSE-HPS, items of the JeffSPLL-HPS were the term “medicine” is used are reworded.

To measure loneliness, the Social and Emotional Loneliness Scale for Adults (SELSA-S), was used. The SELSA-S measures loneliness based in “family”, “romantic”, and “social” dimensions [42]. The SELSA-S (15 items) is answered in a Likert scale from 1 (*strongly disagree*) to 7 (*strongly agree*). Higher scores indicate a greater perception of loneliness. The SELSA-S has shown a high reliability in nurses [2, 43] and in nursing students [34, 44] in Spain and Latin America.

The subjective well-being refers to the emotional and cognitive self-perception of personal life. A Spanish version of the Satisfaction with Life Scale (SWLS) [45] was used as measure of subjective well-being [46, 47]. The scoring of this version is slightly different to the original one developed in English language by Diener et al. [46]. Each item of the Spanish version is scored with a 5-point Likert scale ranging from 1 (*strongly disagree*) to 5 (*strongly agree*), while in the English version each item is scored with a 7-point Likert scale. The Spanish version used in this study has been tested in general population [45] and in healthcare professionals [2, 9] showing a high reliability. A high score in the SWLS is associated with high subjective well-being.

The above psychometric instruments mentioned were validated and tested in previous studies with medical and nursing Peruvian students showing excellent psychometric properties and high reliability [6, 9, 10].

Information regarding age, sex, semester of enrolment, nursing school (public or private), and relationship with parents, were collected through a complementary form. For students’ perception of their relationship with their parents, two separate items were used (one for the mother and one for the father). In each case, respondents were invited to answer to the following statement “the relationship with my (mother/father) is” using a Likert scale from 1 (*there is no relationship*) to 8 (*excellent*).

### Procedures

Questionnaires together with an information letter were distributed in enclosed envelopes to undergraduate students enrolled in the three nursing schools of Cusco. In order to reduce potential bias in students’ responses, the administration of questionnaires were in charge of independent researchers. Students were also informed about the independent nature of this study. Once questionnaires were completed, the participants returned their questionnaires in sealed envelopes following a protocol approved by an independent ethics committee, the “Comité Ético de Investigación de La Rioja” (Ref. CEICLAR-PI-199). There was no potential risk for participants, and anonymity was guaranteed throughout the entire process.

### Statistical analysis

Only the questionnaires with fully completed items were included into the analysis. The reliability was calculated using Cronbach’s alpha coefficient. Values higher than 0.7 were considered satisfactory [48].

As none of the main measures used followed a normal distribution non-parametric tests were performed. For sex (male and female) and nursing school (public and private) variables, a variance analysis (two-way ANOVA) was performed. An interaction effect was also analysed to determine if there were differences in empathy measures defined by a combination of “sex by school”. Finally, effect size was calculated using eta-squared value in order to measure the ratio of variance explained in the dependent variable (empathy) by each of the predictors studied while the others were controlled.

With the purpose of determining possible associations between empathy and the following measures: teamwork and lifelong learning abilities, semester of enrolment, loneliness, subjective well-being, and age, a correlation analysis using Spearman’s coefficient was performed.

Finally, a multilinear regression analysis using variables with significant differences in previous tests was performed. Those variables were tested as predictors of empathy in order to create a model of inference that can explain empathy’s variance. A valid regression model was accepted once the following statistical assumptions were observed: multivariate normality, mean of residuals equal to zero, homogeneity of residuals variance (homoscedasticity), no auto-correlation among residuals, no multicollinearity, and linearity of data. In order to quantify the degree of practical significance of the findings observed in the model obtained, the effect size (Cohen’s *f*^2^) was calculated. An effect size equal to 0.02 was interpreted as small, equal to 0.15 was interpreted as medium, and equal to 0.35 was interpreted as large [49].

All analyses were performed using R statistical software, version 3.6.2 for Windows. The statistical analyses of the data also included *lsr* [50], *multilevel* [51], *apaTables* [52], and *nortest* [53] packages.

## Results

The entire sample included 700 students, corresponding to 68% of the entire population of undergraduate students for the three nursing schools of Cusco city. From this sample, 72 (10%) were men and 628 (90%) women. According to schools, 223 (32%) students were enrolled in the public nursing school, while the others 474 (68%) were enrolled in the two private nursing schools. The mean age was 23 (Mdn=22) with a range from 18 to 57 years old (*SD*=6; IQR=7). According to academic achievement (measured by semester enrolled), the range of semesters covered in the entire sample corresponded with the complete undergraduate program of nursing (10 semesters) that is offered in Peruvian nursing schools. All instruments used showed adequate psychometric properties measured by Cronbach coefficients higher than 0.70 in all cases (see Table 1).

**Table 1:** Descriptive Statistics and Cronbach’s alpha coefficients

	JSE-HPS^a^	JSAPNC^b^	JeffSPLL-HPS^c^	SELSA-S^d^	SWLS^e^
*n*	687	690	690	685	696
Global scores					
Possible Range	20-140	15-60	14-56	15-105	5-25
Actual Range	38-138	15-60	15-56	15-96	5-25
Mean (M)	102.02	49.98	43.93	47.18	18.05
Median (Mdn)	104	52	45	47	19
Interquartile range (IQR)	25	9	8	22	7
Standard Deviation (*SD*)	17.85	8.90	7.64	15.24	5.80
Cronbach’s alpha	0.84	0.91	0.88	0.77	0.82

^a^ Jefferson Scale of Empathy

^b^ Jefferson Scale of Attitudes toward Physician-Nurse Collaboration

^c^ Jefferson Scale of Lifelong Learning

^d^ Social and Emotional Loneliness Scale for Adults

^e^ Satisfaction with Life Scale

Regarding the first objective, results of the two-way ANOVA showed differences in empathy global scores according to “sex” and “nursing school” variables, and also in the interaction of “sex by nursing school”. Female students (M=102.80; Mdn=104; *SD*=17.74) and students enrolled in the public school (M=107.10; Mdn=111; *SD*=19.45) reported higher scores on empathy in comparison with male students (M=95.17; Mdn=97; *SD*=17.47) and with students enrolled in private nursing schools (M=99.56; Mdn=99.50; *SD*=16.55), respectively. Furthermore, the comparison of the interaction between sex and university showed female and male students enrolled in the public school presented the highest and the lowest empathy scores, respectively. The size effect of these three variables in the variance of empathy was small in the case of “sex” (η_p_^2^ = 0.01), and between small and medium in the case of “nursing school” (η_p_^2^ = 0.04) and in the interaction of “sex by nursing school” (η_p_^2^ = 0.02) variables (see Table 2).

**Table 2:** Two-Way ANOVA for empathy in undergraduate nursing students (*n*=700)

Source of variation	*F*(_1,680_)	η^2^	η_p_^2^	*p*
**Main Effects**				
Sex (men vs. women)	9.51	0.01	0.01	0.002
Nursing school (public vs. private)	25.82	0.04	0.04	<0.001
**Two-Way Interaction**				
Sex-Nursing school	15.04	0.02	0.02	<0.001

* F*, F value; η^2^, Eta-squared; η_p_^2^, Eta-partial-square; *p*, p-Value

With regard to the second objective, correlation analyses were performed showing a positive relation between empathy and the following variables: teamwork (ρ=+0.59; *p<*0.001), lifelong learning (ρ=+0.39; *p<*0.001), and having a positive relationship with the mother (ρ=+0.08; *p=*0.03). On the contrary, a negative association was observed between empathy and loneliness (ρ=–0.41; *p<*0.001), and empathy and age (ρ=–0.15; *p<*0.001). Neither academic achievement (ρ=+0.06; *p=*0.14), having a positive relation with the father (ρ=+0.06; *p=*0.13), or subjective well-being (ρ=+0.01; *p*=0.72) showed a significant association with empathy’s global measures, as is shown in Table 3.

**Table 3:** Spearman’s coefficients between empathy (JSE-HPS) and variables with possible role of influence

Variables	ρ	*p*
*Professionalism competencies*		
Teamwork (JSAPNC^a^)	+0.59	<0.001
Lifelong learning (JeffSPLL-HPS^b^)	+0.39	<0.001
*Subjective well-being*		
Life satisfaction (SWLS^c^)	+0.01	0.72
*Relationship with parents*		
Positive mother’s relationship	+0.08	0.03
Positive father’s relationship	+0.06	0.13
*Nursing studies*		
Semester of enrolment	+0.06	0.14
*Loneliness* (SELSA-S^d^)		
Global measure	–0.41	<0.001
Romantic dimension	–0.02	0.67
Social dimension	–0.43	<0.001
Family dimension	–0.45	<0.001
*Age*	–0.15	<0.001

^a^ Jefferson Scale of Attitudes toward Physician-Nurse Collaboration

^b^ Jefferson Scale of Lifelong Learning

^c^ Satisfaction with Life Scale

^d^ Social and Emotional Loneliness Scale for Adults

Based in the above-observed outcomes, a multiple linear regression analysis was carried out in the entire sample (third objective). This analysis produced a model that explained 53% of the variability of the JSE measurement (R^2^-adjusted=0.53; *F*_(6,652)_=122.50; *p<*0.001), with a very large effect size (Cohen-*f*^2^=1.13). According to this model, teamwork abilities, loneliness, subjective well-being, age, sex (female), and nursing school (public), appeared as influencing factors in the development of empathy in patient care. Teamwork abilities, being a female student, and studying in a public nursing school, showed a positive linear relationship with empathy. On the contrary, loneliness, subjective well-being and age, showed a negative influence in the variability of empathy. A summary of this analysis is shown in Table 4.

**Table 4:** A multiple regression model for global scores of the JSE-HPS in nursing students

Predictors	β	*SE*	*t*	*p*
Teamwork (JSAPNC^a^)	+1.16	0.06	+18.81	<0.001
Loneliness (SELSA-S^b^)	–0.27	0.04	–6.90	<0.001
Satisfaction with life (SWLS^c^)	–0.31	0.12	–2.64	0.009
Age	–0.19	0.09	–2.23	0.03
Sex [women]	+4.27	1.56	+2.73	0.007
Nursing school [public]	+3.52	1.11	+3.19	0.002

β, beta coefficient; *SE*, standard error; *t*, *t*–experimental; *p*, *p*–value

^a^ Jefferson Scale of Attitudes toward Physician-Nurse Collaboration

^b^ Social and Emotional Loneliness Scale for Adults

^c^ Satisfaction with Life Scale

This model complied with all the conditions necessary for statistic inference: assumptions of normality of residuals, homogeneity of residuals variance, linearity, no auto-correlation and no multicollinearity. In addition, separate linear regression analyses were performed using teamwork, loneliness, lifelong learning, and life satisfaction (subjective well-being) as dependent variables while all the others were used as potential predictors. These analyses were performed in order to explore whether these variables were sensitive to the influence of empathy and other variables collected. However, none of the models obtained fulfilled all conditions for statistical inference.

**Fig. 1:**
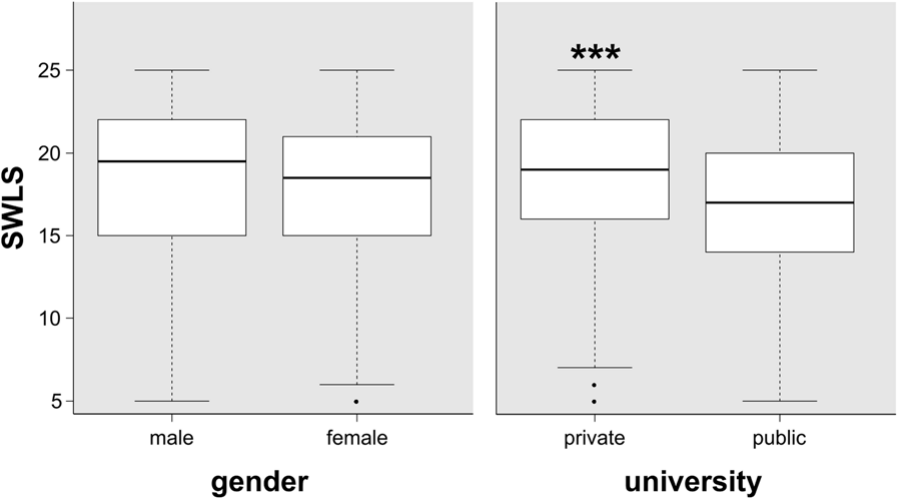
Scores on Satisfaction with Life scale (SWLS) by gender and university; *** *p<*0.001

Finally, with the purpose of having a better understanding of the subjective well-being’s role, this variable was compared by university and sex. Differences appeared in the first case (*p<*0.001), but not in the second one (*p=*0.60), confirming a different pattern in the self-perception of personal life according to nursing school (Fig. 1).

## Discussion

The JSE-HPS showed adequate psychometric properties with Cronbach’s alpha coefficient higher than the international recommendation of 0.70 set by the American Educational Research Association [48]. Results observed in the present study support the use of the Spanish version of the JSE-HPS as a consistent measure of empathy in Peruvian nursing students. These findings are also similar to those previously reported in nursing students from different cultural contexts such as the United States [54, 55], Mexico [35], Italy [27], Spain [44], Peru [9, 34], or Albania [26].

Regarding the first objective, our findings confirmed differences in empathy measurements in nursing students by sex, nursing school and the interaction of both. These findings, regarding sex, are in consonance with previous studies performed in other cultural settings in nursing students and other non-medicine healthcare students where female students showed higher scores on empathy in comparison with their male peers [26, 27, 55–57]. On the other hand, differences observed by nursing school (public vs. private) suggest that, in the absence of a targeted training in empathy, the social environment surrounding the university acquires an important role of influence in the development of empathy in nursing students. This finding brings evidence supporting the idea, proposed by Susanne Täuber, that social environments influence human relationships and social interactions [58]. Elitism, social stereotypes and racial bias, appear as possible manners of this influence. Elitism: In Peru, similarly to other countries, studying in a private university implies an important economic investment for students and their families, not always accessible for everyone. Elitism among students enrolled in these institutions is a social consequence derived from it. In the US, this elitism has been characterized as an influencing factor in the lack of altruist and lower scores in empathy measures in American medical students enrolled in high-ranked institutions [59, 60]. Social stereotypes and racial bias: Social influence can be also strengthening by the effect of cultural and racial stereotypes that are still dominant in the Peruvian society [61]. Indeed, studies in social psychology and neurosciences have revealed that empathic responses may be reduced by social stereotypes and racial bias [62, 63]. Findings observed from the comparison analysis of the scores of the Satisfaction with Life Scale by university groups may reinforce this interpretation.

The second objective was to determine the type of association between empathy and the other variables measured. Empathy showed a positive correlation with measures of teamwork and lifelong learning. These findings bring evidence supporting the theory that these three competences are specific elements of a common construct: professionalism [64]. Furthermore, this finding is in consonance with others reported in Mexico, where a positive association between empathy and teamwork measures was observed in students of nursing [35]. On the contrary, empathy showed an inverse correlation with loneliness measures and age. These findings, in the case of loneliness, are in consonance with others recently reported in Chilean nurses [2] and Spanish nursing students [44]. Age, but not semester of enrolment, showed an inverse association with empathy indicating that this association was due to the students’ age and not by the semester in which the student was attending. Taking this into account, this association may be consequence of the social environment, previously described. In this sense, it is possible that older students become less idealistic and carry more social prejudgments than their younger peers, and this difference is reflected in a lower score in empathy.

Finally, the third objective was to characterize variables as predictors of empathy’s measurements. A linear regression analysis confirmed preliminary findings indicating that teamwork, sex (being a female student) and studying in a public nursing school, are positive predictors of empathy. On the contrary, age, loneliness and subjective well-being appeared as negative predictors of empathy. It is not surprising that subjective well-being plays a negative role of influence in the development of empathy after taking into consideration the differences observed when private and public university groups were compared. In the absence of a targeted training on empathy, it is possible that students enrolled in private universities have less personal resources for empathizing with poor patients. On the contrary, students from public universities probably have more personal resources at the moment to understand and communicate with patients who have to straggling with economic issues related to their treatments.

## Limitations and strengths

To appreciate the findings of this study, some limitations require consideration. First, the design of the study was a cross-sectional self-reporting questionnaire. Self-reporting could lead to response bias or social-desirability bias. Second, analyses are based upon a convenient sample of nursing students from one Peruvian region. And third, loneliness, one of the variables measured, is described as a multiple dimensional concept that can be analysed as a global construct or as separate domains. However, inference analysis performed in this study allowed only a global characterization of loneliness since the models did not fit with all necessary conditions when using separate domains.

On the contrary, the strengths of this study are: the large sample size, which provides great statistical power to analyse the current level of knowledge and elements measured; and the good psychometric properties of the instruments, The sample was composed by nursing students (both from public and private universities) enrolled in schools without targeted educative activities focused to the improvement of empathy, offering an unique opportunity to analyse the real effect that non-academic factors play in the early development of this ability. Another strength is related to the fact that Cusco city has a significant relevance in other cultural contexts, due to its multicultural and multilingual social structure.

### Implications

Empathy is a core competence in nursing. To understand the main factors influencing empathy, which are not directly related with formal curricula, is highly important for educators. Findings observed in this study bring valuable information of non-academic elements that are influencing the empathic ability of nursing students. This knowledge acquires more relevance under current circumstances of the pandemic since many nursing schools are adapting their curricula to e-learning or blended methodologies. This drastic change has important implications in the training of empathic abilities that require intensive social contact. Staying at home privileges the influence that other aspects, associated to the social environments (such as family) and personal resources, have in the early development of this ability. Future research should explore nursing students’ behaviour regarding communication and understanding abilities associated with empathy after the pandemic is over in order to determine suitable methodological strategies for its enhancement in safety conditions.

## Conclusions

Findings have indicated that, in the absence of targeted training on empathy, this ability is sensitive to the influence of personal characteristics and of the social and family environments. Genetic predisposition associated with gender, personal life experience, social skills, and loneliness appear as important factors of influence associated with personal characteristics. On the other hand, the social environment and the family appear as two important influencing factors in the development of empathy.

## Supplementary information

**Additional file 1**

## Data Availability

The data generated and analysed during this study are included in this published article as a supplementary file (dataset.xlsx).

## Abbreviations

JSE-HPS: Healthcare student’s version of the JSE
JSAPNC: Jefferson Scale of Attitudes toward Physician-Nurse Collaboration
JeffSPLL-HPS: Healthcare student’s version of the JeffSPLL
SELSA-S: Short version of the Social and Emotional Loneliness Scale for Adults
SWLS: Satisfaction with Life Scale
